# Survival strategies for choosing the right postdoc position

**DOI:** 10.1186/gb4163

**Published:** 2014-03-10

**Authors:** Duncan T Odom

**Affiliations:** 1 University of Cambridge, Cancer Research UK Cambridge Institute, Robinson Way, Cambridge, CB2 0RE, UK

## 

You are completing your PhD (at last!), and might be asking yourself, ‘What do I have to accomplish as a postdoc if I want to get a faculty job at one of the top 25% of research universities?’ This article is a follow up to last year’s essay on picking a graduate studentship, and here I lay out the advice I give to young scientists who have determined that they want to make the next step towards a research faculty post (however, see Box 1).

See related comment on picking a graduate studentship, http://genomebiology.com/2013/14/4/114

## What is success?

If your career goal is to be a contender for a top-tier research group leader position in this day and age, the goal of a postdoctoral fellowship is profoundly different than that of a studentship. In a graduate studentship, solid success is a couple of good, mid-level peer-reviewed papers. In a postdoctoral fellowship, success would be counted as having published leading articles (note the plural) in top-tier journals. There are obviously exceptions to this, but they are exactly that: exceptional.

The most reliable way to be seriously looked at as a faculty candidate is to have cold, hard proof of your research caliber, meaning one or two major, highest impact stories as first (or co-first) author, followed by one to three additional stories as first (or co-first) author in field-leading journals such as *Molecular Cell* or *Genome Biology*. The first triage that a typical search committee does is to remove all CVs that do not have any publications, followed by those that have no substantive first author publications. Papers listed as ‘in preparation’ are generally ignored. Committees almost always look favorably on candidate experience in multiple scientific environments, and some committees may also consider strong expertise (read: good publications) in multiple diverse fields to be attractive.

## When to choose a host laboratory for your postdoc?

Answer: 12 months before you want to start a postdoc.

With that in mind as a graduate student, you must plan on where to go and what to do a full year in advance. The guidelines I gave last year for where to go as a graduate student produce a reasonably large and diverse set of prospective host laboratories. These include many nationally prominent groups who rarely publish papers in journals-with-one-word-titles, and are often awesome training environments whose graduate students go on to great careers. The postdocs in these labs, however, rarely can compete directly in the job market with those that have the biggest name journal articles - with or without demi-god advisors. The faculty job market is just too competitive.

It is imperative that you contact potential advisors twelve (12) months in advance (yes, I have said this repeatedly), because all the best labs are *always* oversubscribed and need lots of lead time for a new hire. Besides, there will almost certainly be a (serious) interview as well, for the same reason. It also gives you, as a postdoc candidate, sufficient lead time to both informally inquire in your extended social network about the host lab, and to obtain your own funding, which may increase your perceived value to any host lab immediately - you were a free hire. Some labs require you to get outside funding, fullstop: it is imperative that you directly ask what funding you are expected to obtain, and when. And of course, don’t forget that many countries have bureaucratically slow and occasionally xenophobically paranoid visa systems to navigate, even for citizens of economically more-developed countries.

## Characteristics to look for in a host lab

Now, who do you look for? This is where the decisions a graduate student would make are a bit different. Postdocs unfortunately require high impact papers, so you must identify a laboratory where there is a consistent and conscious commitment to do everything possible to enable and ensure every postdoc to publish these. Evidence for this commitment can be seen simply by looking at how diverse the first-author names are in the prospective host lab’s biggest papers, combined with frequency of publications.

Obviously, there are never guarantees in life, but if a prospective advisor flatly refuses to commit to doing their best to help your career or says ‘it’s mostly up to the concerned postdoc’s work ethic’ - well, you should probably find a different postdoc home. It is rarely said overtly, but this stage of your career is a mutual and two-way agreement: you give your most productive years, and the advisor in return will break their back to make sure *everyone* wins (read: publishes outstanding stories).

## Lab size

If you are high caliber as a postdoctoral scientist, then you are not going to need nearly as much attention as when you were first starting, so you can choose a host lab head who is a bit more hands-off. The default option often means an established and prominent lab in your scientific area. However, the size of the lab is as crucial as when you were a graduate student: it is my personal opinion that (except for rare cases) in excess of four, maybe five, postdocs in a typical laboratory, in genomics at least, is a recipe for an unhealthy environment.

First of all, ideally, everyone in a lab knows everything else that other folks are doing to ensure scientific cross-fertilization and criticism, as well as prevent intra-lab competition. More than five postdocs (in a group of, say, ten total workers) means that this goal is not realistic. Secondly, as a postdoc, you need to be seriously considering what your own special niche will be once you leave, and how to reduce the chances of your creative ideas being stolen out from under you. In a huge Darwinian lab, everyone is aggressively looking for high-impact projects - *any* high impact project. Three years into your new faculty job, just as you are writing up your first story, you may find the cherished idea you were collegially discussing before your departure comes out in a big name journal, led by someone from (or, worse, even still in) your old lab, just as you are writing it up yourself with your very first postdoc (note: I have witnessed this exact situation happen to numerous new faculty). In a case like this, some former bosses can argue, with plausible deniability, that they had no idea they were scooping you - because they have too many people to keep track of.

Problems can occur in the other direction with an established lab that is too small. If a lab has less than about seven active researchers, it can be hard to have sufficient diversity of co-workers to drive a broad scientific programme. However, this certainly does not apply to starting up, junior group leader labs.

Regardless, established labs should also be evaluated based on their placement record for prior postdocs and the political and scientific connections to place folks in the future, as well as how fairly and evenly resources are distributed among projects. You can always ask around for good information from neighboring labs: people love to gossip.

## Lab stage

Most of the above has been directed at how to find a good established lab. However, folks who have just started as junior group leaders at leading research institutes are very often overlooked, and are about the best and safest bets out there - if you can get on board early enough. By the time they win prestigious ‘Young Investigator’ type prizes, it may be too late. Most searching grad students think these labs are high risk as a postdoc, but if your goal is to survive with a highest impact paper, well, let’s just say that these group leaders’ interests and your interests are almost perfectly aligned. In contrast, does Prof. Nobel-Prize care about your first (but their 17^th^*Cell* paper)? Maybe, but nowhere near as much as Assistant Prof. Small-Fry, who cares a whole awful lot!

The main risk of this strategy is that really junior groups in the first four years can be micromanaged as well as very tense places, as everyone worries about the lab’s survival. There are also almost certainly going to be serious birth pains and discomfort when you leave, as junior group leaders have to be more concerned than established labs about what can be taken away by departing postdocs. On the other hand, they are probably less likely than established labs to ‘inadvertently’ steal your ideas, as they just faced this problem from the other side and will have more compassion.

## Leave your comfort zone

Challenge yourself, and don’t limit your search to the place where you have been for the last 10 years (yes, I mean you, Oxbridge, San Diego, Boston!). Having sat on faculty search committees, it is attractive to know that a faculty candidate had the courage to face the world by proverbially moving out of their comfy, rent-free parents’ cellar.

## The future is your responsibility

Regardless of what you do, the choices *you* make in choosing your new lab home are the biggest determinants of your future. Be polite but pressing in your questioning before and during interviews. Ask folks hard questions. Seek gossip about what the lab’s dynamics are. If you discover after your failed postdoc that ‘everyone knew’ that Prof. Nobel-Prize always pits people against each other on the same project, then it is *your* failure that you did not ask around enough before you joined the lab.

In sum, if you don’t have the passion, resiliance and sheer bloodymindedness needed to struggle through all this, it might be safest to find an alternative career that is more supportive and laid back, even if less well remunerated than science. Perhaps art therapy, accountancy or investment banking.

## Box 1: Is a postdoc the right career choice for you?

Dear reader, please know that I cannot and will not defend the justice of the system I am describing; instead, I am attempting to give practical and conservative advice on how to increase your probability of surviving it. My most honest advice is that you should only pursue an academic career if you literally cannot see yourself doing anything else and living a fulfilled life. Academia is training far too many graduate students, instilling ridiculous expectations of a rosy future, and then losing way over half our personnel investments at the faculty job interview stage (see Box 2). If you, for instance, always wanted to make blown glass sculpture, then for your own sanity, escape now! A further caveat to my advice is that it is grounded in the experience of a basic biology/modern genetics laboratory, and other fields may differ in specific details and relevant advice.

## Box 2. Further reading

• Schneider L: **Observations of a frustrated scientist.***LabTimes* 2013, http://www.labtimes-archiv.de/epaper/LT_13_06/#57. Leonid does a great job highlighting the bad behaviours of labs you should try to avoid and crystallizing the bitterness this system engenders. I am slightly more optimistic with my pragmatism: just try to avoid the worst aspects by carefully choosing your path.

• **Supply-side academics.***Nat Neurosci* 2007, **10:**1337. http://www.nature.com/neuro/journal/v10/n11/full/nn1107-1337.html

• Wadman M: **A workforce out of balance.***Nature* 2012, **486:**304. http://www.nature.com/news/a-workforce-out-of-balance-1.10852

• Afonso A: **How academia resembles a drug gang.**http://alexandreafonso.wordpress.com/2013/11/21/how-academia-resembles-a-drug-gang/. A very enjoyable read that, in part, suggests interesting gaming strategies based on geographic movement to sidestep the worst of the market distortions. He has great references, too.

• **The disposable academic.***The Economist* 2010, http://www.economist.com/node/17723223

